# Heme Oxygenase-1-Modified Bone Marrow Mesenchymal Stem Cells Combined with Normothermic Machine Perfusion Repairs Bile Duct Injury in a Rat Model of DCD Liver Transplantation via Activation of Peribiliary Glands through the Wnt Pathway

**DOI:** 10.1155/2021/9935370

**Published:** 2021-07-01

**Authors:** Xuan Tian, Huan Cao, Longlong Wu, Weiping Zheng, Mengshu Yuan, Xiang Li, Hongli Song, Zhongyang Shen

**Affiliations:** ^1^School of Medicine, Nankai University, Tianjin, China; ^2^Tianjin First Central Hospital Clinic Institute, Tianjin Medical University, Tianjin 300070, China; ^3^Department of Organ Transplantation, Tianjin First Central Hospital, School of Medicine, Nankai University, Tianjin 300192, China; ^4^NHC Key Laboratory of Critical Care Medicine, Tianjin 300192, China; ^5^Tianjin Key Laboratory of Organ Transplantation, Tianjin, China; ^6^Key Laboratory of Transplant Medicine, Chinese Academy of Medical Sciences, Tianjin, China

## Abstract

Livers from donors after circulatory death (DCD) are inevitably exposed to a longer warm ischemic period, which might increase the incidence of postoperative bile duct complications. Bone marrow mesenchymal stem cells (BMMSCs) have tissue repair properties. The present study was aimed at exploring the repair effect of heme oxygenase-1- (HO-1-) modified BMMSCs (HO-1/BMMSCs) combined with normothermic machine perfusion (NMP) on bile duct injury after DCD liver transplantation and at revealing the underlying mechanisms. Rat livers were exposed to *in situ* warm ischemia for 30 min; then, NMP was performed through the portal vein for 4 h with BMMSCs, HO-1/BMMSCs, or neither before implantation. Obvious bile duct histological damage and liver functional damage were observed postoperatively. In the group treated with HO-1/BMMSCs combined with NMP (HBP group), liver functions and bile duct histology were improved; meanwhile, cell apoptosis was reduced and cell proliferation was active. A large number of regenerative cells appeared at the injured site, and the defective bile duct epithelium was restored. Dilatation of peribiliary glands (PBGs), proliferation of PBG cells, high expression of vascular endothelial growth factor (VEGF), and increased proportion of bile duct progenitor cells with stem/progenitor cells biomarkers were observed. Blocking Wnt signaling significantly inhibited the repair effect of HO-1/BMMSCs on bile duct injury. In conclusion, HO-1/BMMSCs combined with NMP were relevant to the activation of biliary progenitor cells in PBGs which repaired bile duct injury in DCD liver transplantation via the Wnt signaling pathway. Proliferation and differentiation of PBG cells were involved in the renewal of the injured biliary epithelium.

## 1. Introduction

Liver transplantation is a life-saving therapy for patients with end-stage liver disease; however, organ shortage is currently the biggest obstacle to successful treatment, which directly leads to the death of thousands of patients on waiting lists each year [1]. Many liver transplantation centers currently accept grafts from extended criterion donors (ECD), a large proportion of which are donors after circulatory death (DCD). The characteristic of DCD livers is that they undergo a longer warm ischemic period than livers from donors after brain death (DBD) and are more susceptible to ischemia-reperfusion injury (IRI). Compared with parenchymal hepatic cells, biliary epithelial cells have a lower tolerance to IRI because of a lack of self-protective antioxidants such as catalase and reduced glutathione [2]. Biliary complications are important factors affecting the survival rate and long-term outcome of liver transplantation. The incidence of biliary complications after DCD liver transplantation is between 25 and 60%, while that of DBD liver transplantation is 10 to 30% [3]. Traditional static cold storage can reduce cell metabolism and has a decent preservation effect on grafts from standard criterion donors (SCD); however, it does not work satisfactorily in the preservation of ECD organs, including DCD livers [4]. Therefore, it is important to explore new strategies to improve the quality of DCD livers, reduce postoperative biliary complications, and prolong survival time.

Mesenchymal stem cells (MSCs) have been shown to contribute to tissue repair regeneration through secreting cytokines and extracellular vesicles [5–8]. However, the ability of MSCs to “home” to injured tissue is controversial, because most MSCs are actually trapped in pulmonary capillaries after intravenous administration [5]. Previous studies have shown that normothermic machine perfusion (NMP) could promote engraftment of bone marrow mesenchymal stem cells (BMMSCs) in the liver, and heme oxygenase-1 (HO-1) could prolong the survival of BMMSCs in the injured site [9, 10]. Therefore, in the present study, we aimed to infuse HO-1-modified BMMSCs (HO-1/BMMSCs) into the liver using NMP. During *ex situ* preservation, NMP could maintain a reasonable normal metabolism by providing the liver with oxygen and nutrients, mimicking the physiological environment *in vitro*. Meanwhile, it also improves the utilization efficiency of BMMSCs.

Peribiliary glands (PBGs) along the extrahepatic and large intrahepatic bile ducts contain stem and progenitor cells with pluripotent properties, which would activate, proliferate, and differentiate to repair the damaged bile duct epithelium [11, 12]. Therefore, we aimed to evaluate the repair effect of HO-1/BMMSCs combined with NMP on bile duct IRI in DCD liver transplantation, to observe changes in PBG cells, and to further investigate the underlying mechanisms.

## 2. Materials and Methods

### 2.1. Reagents

The present study used Dulbecco's modified eagle medium (DMEM)/F12 medium (Solarbio, Beijing, China); fetal bovine serum (Biowest, Loire Valley, France); BMMSC surface marker-related antibodies (anti-rat cluster of differentiation 34- (CD34-) fluorescein isothiocyanate (FITC), anti-rat CD29- (integrin subunit beta 1-) phycoerythrin (PE), anti-rat protein tyrosine phosphatase receptor type C- (CD45-) PE, anti-rat CD90- (Thy-1 cell surface antigen-) FITC, anti-rat RT1A- (rat MHC class I antibody-) PE, and anti-rat RT1B- (rat MHC class II antibody-) FITC (BioLegend, San Diego, CA, USA)); adipogenic and osteogenic differentiation medium (Sigma-Aldrich, St. Louis, MO, USA); Oil Red O (Dingguo Changsheng Biotechnology, Beijing, China); von Kossa cell staining kit (Genmed, Shanghai, China); rat green fluorescent protein genomic adenovirus (GFP-Adv, GeneChem, Shanghai, China); mouse antibodies recognizing cystic fibrosis transmembrane conductance regulator (CFTR) (Santa Cruz, Dallas, TX, USA), SRY-box transcription factor 9 (SOX9) (Santa Cruz), Nanog (Santa Cruz), proliferating cell nuclear antigen (PCNA) (Santa Cruz), and Wnt3 (Santa Cruz); rabbit antibodies recognizing *β*-catenin (Santa Cruz), vascular endothelial growth factor (VEGF) (Proteintech, Wuhan, China), and occludin (Proteintech); tight junction protein 1 (TJP1, also known as ZO-1) (Proteintech, Wuhan, China), caspase-3 (Cell Signaling Technology, Danvers, MA, USA), and active *β*-catenin (Cell Signaling Technology); *β*-actin mouse antibodies (Beyotime, Shanghai, China); and XAV-939 (MedChemExpress, Monmouth Junction, NJ USA).

### 2.2. Animals

Male Sprague-Dawley (SD) rats were purchased from the National Institutes for Food and Drug Control (Beijing, China) and reared on a standard laboratory diet with water. Four-week-old rats (weighing about 50 g) were used to isolate BMMSCs, and 8-week-old rats (about 220 g) were used to create the orthotopic liver transplantation (OLT) model. Twenty animals (*n* = 5 in each experimental group) were used for sample collection on the 7th day after OLT, 24 animals (*n* = 6 in each group) were used for survival rate analysis, 20 animals (*n* = 5 in sham group, *n* = 5 at each point in time (1 d, 7 d, 14 d) in the NMP group) were used for biochemical analysis, 3 animals were used for BMMSC frozen section tracking, and 3 animals were used for BMMSC *in vivo* tracking. All animal experiments followed the Guidelines for the Care and Use of Laboratory Animals and were approved by the Ethics Committee of Tianjin First Central Hospital (Permit number: 2016-03-A1).

### 2.3. Isolation and Identification of HO-1/BMMSCs

The isolation, culture, identification, and gene transfection of BMMSCs were carried out as described previously [9, 10]. Bone marrow contents were extracted from the rat femur and tibia, and BMMSCs were obtained by the continuous adhesion method [13]. Adenovirus vectors were used to transfect BMMSCs with constructs expressing the green fluorescent protein (GFP) and/or HO-1. HO-1/BMMSCs were identified by osteogenic and adipogenic differentiation *in vitro*.

### 2.4. DCD Liver Procurement

DCD liver procurement was performed as described previously [14]. Following midline incision, the diaphragm was opened and the thoracic aorta was clipped to induce cardiac arrest after previous heparinization. After 30 minutes of *in situ* warm ischemia, the livers were flushed with cold heparinized saline through the portal vein and then excised and preserved. The portal vein was cannulated to connect to the perfusion machine.

According to different preservation methods of livers, animals were randomly divided into the following groups: the sham group (sham operation), the NMP group (livers were perfused by NMP via the portal vein for 4 h before implantation), the BP group (livers were perfused by NMP combined with BMMSCs via the portal vein for 4 h before implantation), the HBP group (livers were perfused by NMP combined with HO-1/BMMSCs via the portal vein for 4 h before implantation), and the XAV group (livers were perfused by NMP combined with HO-1/BMMSCs via the portal vein for 4 h before implantation, and intraperitoneal injection of XAV-939 was administered continuously for 7 days postoperatively at 5 mg/kg/d). XAV-939 is an inhibitor of the Wnt/*β*-catenin signal pathway, which degrades *β*-catenin by stabilizing Axin. Each group included five animals.

### 2.5. Normothermic Machine Perfusion

The composition of the NMP system was the same as reported previously [10, 14] (Fig. S1). The total volume of perfusate was 80 mL. The components were as follows: 60 mL of DMEM/F 12 medium containing 20% fetal bovine serum and 20 mL of rat blood (the hematocrit of the perfusate was 10–15%). The following drugs were added: penicillin 4000–8000 U (50–8000 U/mL), streptomycin 4000–8000 *μ*g (50–100 *μ*g/mL), heparin 200–400 U (2–5 U/mL), dexamethasone 80–200 *μ*g (1–2.5 *μ*g/mL), and insulin 40–80 U (0.5–1 U/mL).

After removal from the donors, livers were connected to the NMP system through a stent in the portal vein (single cycle perfusion). Machine perfusion was performed at a flow rate of 10–15 mL/min for 4 hours, and the perfusion pressure was maintained at 900–1400 Pa. Oxygen tension in the portal vein was maintained at 75–90 mmHg (10000–12000 Pa). The perfusate was oxygenated using a membrane oxygenator and maintained at 37–38°C. The bile duct was intubated to collect bile. BMMSC or HO-1/BMMSC single cell suspensions (containing 1–3 × 10^7^ cells) were infused by syringe injection via the portal vein cannula.

### 2.6. Liver Transplantation Model

Rat nonarterialized orthotopic liver transplantation was performed using the two-cuff technique [15]. The suprahepatic inferior vena cava was sutured end-to-end. The portal vein and the infrahepatic vena cava were anastomosed using the cuff technique. The bile duct was anastomosed end-to-end through a stent. All operations were performed by the same doctor, and the anhepatic period was 21 ± 2 minutes.

### 2.7. Tracking of BMMSCs in Living Rats

BMMSCs or HO-1/BMMSCs transfected with adenovirus vectors expressing GFP (GFP/Ad) were injected into livers via NMP and then traced *in vivo* on the 1^st^ day after OLT using a small animal *in vivo* imaging system (Maestro EX in-vivo fluorescence imaging system (Cambridge Research & Instrumentation, Inc. (CRi), Woburn, MA, USA)) on the first day after liver transplantation.

### 2.8. Biochemical Examination

Animals were sacrificed on the 1^st^, 7^th^, and 14^th^ day after liver transplantation, and serum samples were collected from the inferior vena cava for biochemical analysis (*n* = 5 per group at each point in time). The levels of serum alanine aminotransferase (ALT), aspartate aminotransferase (AST), gamma-glutamyl transpeptidase (GGT), and total bilirubin (TBil) were detected using an automatic biochemical analyzer (Cobas 800; Roche Diagnostics, Basel, Switzerland).

### 2.9. Transmission Electron Microscopy

Animals were sacrificed on the 7^th^ day after liver transplantation for sample collection. Fresh liver tissue was cut into 1 × 1 × 2 mm^3^ samples, fixed in 2.5% glutaraldehyde, embedded in epoxy resin, and sliced into ultrathin sections. The tissue ultrastructure was observed using a transmission electron microscope (HT7800, Hitachi, Tokyo, Japan).

### 2.10. Histology

Animals (*n* = 5 per group) were sacrificed on the 7^th^ day after liver transplantation for histological examination. The common bile ducts were resected from the bifurcation of the left and right hepatic ducts to the anastomosis of the bile ducts. The liver tissue was cut into 2 × 2 × 1 cm^3^ samples. The tissue was fixed in 10% formalin solution and then embedded in paraffin. Serial 3 *μ*m sections were obtained and stained with hematoxylin-eosin (HE).

### 2.11. Immunohistochemistry

Animals were sacrificed on the 7^th^ day after liver transplantation for sample collection. Sections were deparaffinized using dimethylbenzene and hydrated with a gradient alcohol series. Antigen retrieval was performed by heating in EDTA (pH 9.0) in a microwave for 15 minutes. The tissue slides were then incubated in H_2_O_2_ for 30 minutes to block endogenous peroxidase activity and further blocked with 5% normal goat serum. The slides were incubated overnight at 4°C with primary antibodies recognizing PCNA (1 : 300), caspase-3 (1 : 500), Nanog (1 : 300), and SOX9 (1 : 300). Afterwards, the slides were incubated with biotinylated secondary antibodies at room temperature for 30 minutes and streptavidin-horseradish peroxidase (HRP) at room temperature for 30 minutes. Finally, the slides were stained with diaminobenzidine (DAB) and counterstained with hematoxylin. The proportion of positive cells was calculated as the percentage of positive cells to the total number of cells in PBGs. All histological analyses were performed blindly by manual counting in 20 random fields (10x magnification).

### 2.12. Immunofluorescence Assays

Animals were sacrificed on the 7^th^ day after liver transplantation for sample collection. For immunofluorescence, slides underwent deparaffinization, hydration, antigen retrieval, and blocking as in Section 2.11. The slides were then incubated overnight at 4°C with primary antibodies recognizing *β*-catenin (1 : 300), VEGF (1 : 300), and Nanog (1 : 300) and then labeled with isotype-specific secondary antibodies at room temperature for 1 hour. Finally, the slides were counterstained 4′,6-diamidino-2-phenylindole (DAPI) and observed by fluorescence microscopy.

### 2.13. Quantitative Real-Time Reverse Transcription Polymerase Chain Reaction (qRT-PCR)

Total RNA was extracted using the TRIzol reagent (Takara Biotechnology, Shiga, Japan), and cDNA was synthesized using a cDNA reverse transcription kit (Takara Biotechnology) according to the manufacturer's instructions. The cDNA was then used as a template for the quantitative real-time PCR (qPCR) step using the primer sequences listed in Table S1 and TB Green Premix Ex Taq (Takara Biotechnology). *Actb* (encoding *β*-actin) was used as the reference to correct the expression level (ΔCt) and compared with the baseline (ΔΔCt). Results were presented as fold change (2^−ΔΔCt^) [16].

### 2.14. Western Blotting

Total protein was extracted using radioimmunoprecipitation assay (RIPA) buffer, and phenylmethanesulfonyl fluoride (PMSF) was added to prevent protein degradation. The total protein concentration was determined using a bicinchoninic acid assay (BCA). After separation with sodium dodecyl sulfate-polyacrylamide gel electrophoresis (SDS-PAGE), proteins were transferred to a polyvinylidene fluoride (PVDF) membrane and blocked with 5% skimmed milk for 1 hour. The membranes were then incubated overnight at 4°C with primary antibodies recognizing VEGF (1 : 1000), PCNA (1 : 1000), caspase-3 (1 : 1000), Nanog (1 : 500), SOX9 (1 : 1000), CFTR (1 : 500), *β*-catenin (1 : 3000), active *β*-catenin (1 : 500), and *β*-actin (1 : 3000). Membranes were washed with TBST buffer (Tris buffer with sodium chloride and Tween 20) and incubated with the corresponding labeled secondary antibodies (1 : 2000) at room temperature for 1 hour. The ChemiDoc XRS+ system (Bio-Rad, Hercules, CA, USA) was used for detection, and the ImageJ 7.0 software (National Institutes of Health, Bethesda, MD, USA) was used to analyze the gray values and calculate the relative protein expression level.

### 2.15. Statistical Analysis

All statistical analyses were carried out using SPSS 23 (IBM Corp., Armonk, NY, USA). Normally distributed data are indicated as the mean ± standard deviation and were assessed using one-way analysis of variance or a two-sample Student's *t* test. *P* < 0.05 was considered to be statistically significant. GraphPad Prism 8 (GraphPad Software Inc., San Diego, CA, USA) was used for the graphical presentation of the data.

## 3. Results

### 3.1. Identification of BMMSCs and HO-1/BMMSCs

HO-1/BMMSCs have a shuttle-shaped appearance arranged in a swirl, with typical morphological characteristics of mesenchymal stem cells (Fig. S2A). Under different induction conditions, HO-1/BMMSCs could differentiate into adipocytes or osteoblasts (Fig. S2 B,C), indicating that they have pluripotent potential. HO-1/BMMSCs expressed specific biomarkers of mesenchymal stem cells, and flow cytometry showed that more than 99% of the cells were positive for CD29, CD90, and RT1-A, but negative for CD34, CD45, and RT1-B (Fig. S2 D-F).

After transfection with HO-1/Ad, BMMSCs showed no changes to their biological characteristics, while the expression of HO-1 increased significantly (Fig. S2 G,H,I).

### 3.2. HO-1 Combined with NMP Increased the Engraftment of BMMSCs in Implanted Livers

**Figure 1:**
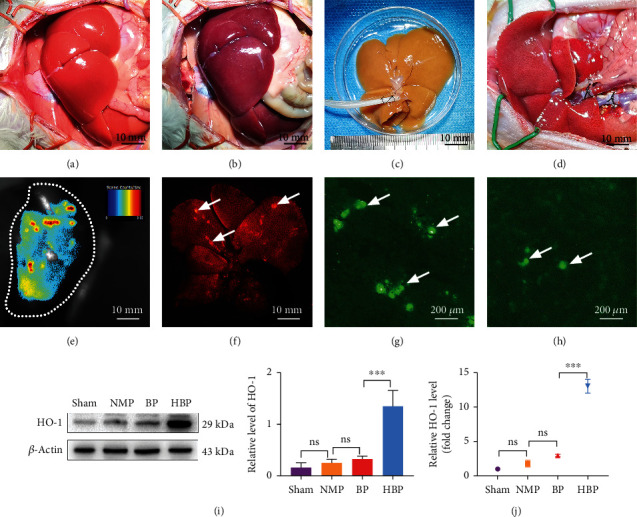
Process of DCD liver transplantation and engraftment of bone marrow mesenchymal stem cells in implanted livers. (a) Normal liver with a pink, homogeneous appearance. (b) After 30 minutes of warm ischemia, the liver was congested with heterogeneous dark purple areas. (c) After NMP for 4 h, the liver was yellowish-brown without congestion. (d) The blood flow of the liver was restored after implantation, and the appearance of liver basically returned to normal. *In vivo* (e) and *in vitro* (f) imaging showing engraftment of BMMSCs in liver. Frozen sections showing that the survival rate of HO-1/BMMSCs (g) in the liver was higher than that of BMMSCs (h). Western blotting (i) and PCR (j) showing high HO-1 expression in the HBP group at 7 days after liver transplantation (*n* = 3). ^∗^*P* < 0.05, ^∗∗^*P* < 0.01, and ^∗∗∗^*P* < 0.001. DCD: donors after circulatory death; NMP: normothermic machine perfusion; BMMSCs: bone marrow mesenchymal stem cells; HO-1: heme oxygenase-1; HBP: HO-1/BMMSCs plus NMP.

Compared with normal livers, DCD livers (warm ischemia for 30 minutes) were dark purple with patchy ecchymosis. After NMP, the livers returned to a homogeneous yellowish appearance without edema (Figures 1(a)–1(c)). A few minutes after implantation, livers were restored with blood flow and showed a reddish appearance (Figure 1(d)). HO-1/BMMSCs transfected with GFP injected by NMP could be observed *in vivo* in livers on the 1st day after implantation (Figures 1(e) and 1(f)). The presence of BMMSCs could be detected in liver frozen sections on the 7th day after implantation under a fluorescence microscope, and more HO-1/BMMSCs had engrafted than BMMSCs (Figures 1(g) and 1(h)). Western blotting on the 7th day after implantation showed that the expression of HO-1 in the HBP group was significantly higher than that in the other groups (Figures 1(i) and 1(j)), indicating that HO-1 transfection could improve the survival rate of BMMSCs in the implanted liver.

### 3.3. HO-1/BMMSCs Combined with NMP Prolonged the Survival of DCD Liver Recipients and Improved Liver Function

**Figure 2:**
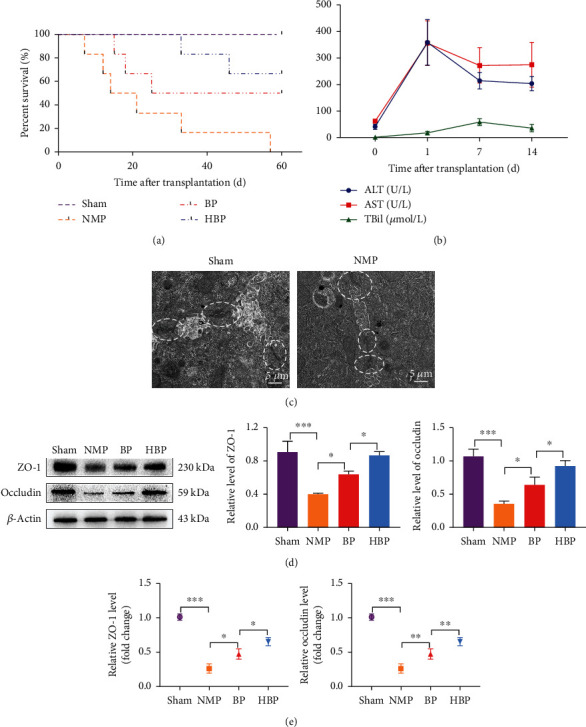
Survival, liver function, and bile ductule ultrastructure of DCD liver transplantation recipients. (a) Survival curve of DCD liver transplantation recipients (*n* = 6 per group). HO-1/BMMSCs combined with NMP improved the survival time of the recipients. Median survival time of sham, NMP, BP, and HBP groups was >60 days, 17.5 days, 42.5 days, and >60 days, respectively. (b) Liver functions of the NMP group on the 1st, 7th, and 14th day postoperatively (*n* = 5). ALT and AST reached their peak values on the postoperative day 1 and TBil on the 7th day. After the 7th postoperative day, all indexes gradually leveled off. (c) On the 7th day postoperatively, transmission electron microscopy (TEM) showed destruction of tight junctions (white ellipses) of bile ductules after liver transplantation compared with the normal liver. Western blotting (d) and PCR (e) showed that expression levels of tight junction proteins (ZO-1 and occludin) in the HBP group were higher than those in the BP and NMP groups (*n* = 3). ^∗^*P* < 0.05, ^∗∗^*P* < 0.01, and ^∗∗∗^*P* < 0.001. DCD: donors after circulatory death; NMP: normothermic machine perfusion; BMMSCs: bone marrow mesenchymal stem cells; HO-1: heme oxygenase-1; HBP: HO-1/BMMSCs plus NMP; BP: BMMSCs plus NMP; ALT: alanine aminotransferase; AST: aspartate aminotransferase; TBil: total bilirubin.

The survival time of the recipients in the NMP group was the shortest. HO-1/BMMSCs combined with NMP prolonged the survival time significantly, and the effect was more obvious than that of BMMSCs combined with NMP (Figure 2(a)).

In the NMP group, indices of liver function (ALT, AST, GGT, and TBil) increased rapidly on the first day postoperatively and gradually decreased after the 7th day postoperatively (Figure 2(b)). Tight junctions (as indicted by ZO-1 and occludin levels) in bile ductules were obviously destroyed compared with that before transplantation (Figure 2(c)), which was the anatomical basis of bilirubin leakage back into the bloodstream. On the 7th day postoperatively, HO-1/BMMSCs could significantly protect tight junctions (as indicted by ZO-1 and occludin levels) from destruction (Figures 2(d) and 2(e)).

**Figure 3:**
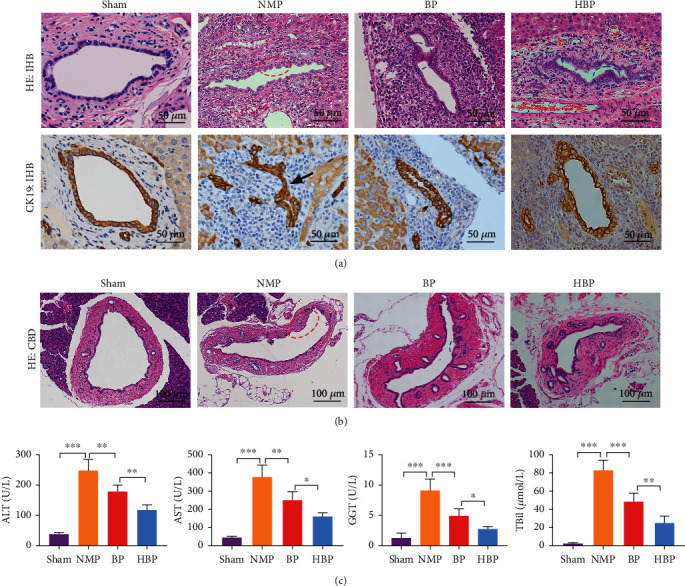
Changes of bile duct histology and liver function. (a, b) HE staining showing intact bile duct morphology and continuous epithelium in the sham group, while continuity of bile duct epithelium was destroyed (red ellipse) and immunohistochemistry of CK19 showed occlusion of large intrahepatic bile duct in the NMP group (black arrow). In the BP and HBP groups, a large number of regenerative biliary cells (blue ellipse) were observed, and continuity of the bile duct epithelium was restored. (c) Liver function in each group 7 days after operation (*n* = 5). ALT, AST, GGT, and TBil in the sham group were 38.96 ± 5.09 U/L, 44.16 ± 7.67 U/L, 2.50 ± 0.70 U/L, and 1.28 ± 0.77 *μ*mol/L, respectively, while those in the NMP group were significantly increased (248.80 ± 36.78 U/L, 375.30 ± 66.91 U/L, 82.56 ± 11.34 U/L, and 9.10 ± 1.90 *μ*mol/L, respectively). Compared with the NMP group, the liver functions in the BP group (179.50 ± 21.42 U/L, 250.60 ± 44.92 U/L, 48.36 ± 9.21 U/L, and 4.92 ± 1.18 *μ*mol/L, respectively) and HBP group (118.30 ± 16.95 U/L, 161.20 ± 18.98 U/L, 24.72 ± 7.54 U/L, and 2.74 ± 0.39 *μ*mol/L, respectively) were improved significantly. CBD: common bile duct; IHBD: intrahepatic bile duct. ^∗^*P* < 0.05, ^∗∗^*P* < 0.01, ^∗∗∗^*P* < 0.001. HE: hematoxylin and eosin; CK19: cytokeratin 19; NMP: normothermic machine perfusion; HBP: HO-1/BMMSCs plus NMP; BP: BMMSCs plus NMP; ALT: alanine aminotransferase; AST: aspartate aminotransferase; GGT: gamma-glutamyl transpeptidase; TBil: total bilirubin.

Histology showed severe tissue damage in the common bile duct (CBD) and intrahepatic bile duct (IHBD) in the NMP group, with discontinuity of the bile duct epithelium, loss of the bile duct epithelium in the lumen, interstitial necrosis of the wall, inflammatory cell infiltration, and few regenerative biliary cells (Figure 3(a)). Cytokeratin 19 (CK19) immunohistochemical staining showed occlusion of the IHBD lumen (Figure 3(a)). By contrast, most of the bile duct epithelium in the HBP group displayed restored continuity without interruption, and a large number of regenerative cells in the bile duct lumen were observed, which was improved compared with that in the BP group (Figures 3(a) and 3(b)). Levels of serum ALT, AST, GGT, and TBil in the HBP group were significantly lower than those in the other groups (Figure 3(c)), indicating that HO-1/BMMSCs combined with NMP could effectively improve liver function after liver transplantation.

### 3.4. PBGs Were Involved in the Regeneration of Biliary Cells and the Repair of Bile Duct Injury

**Figure 4:**
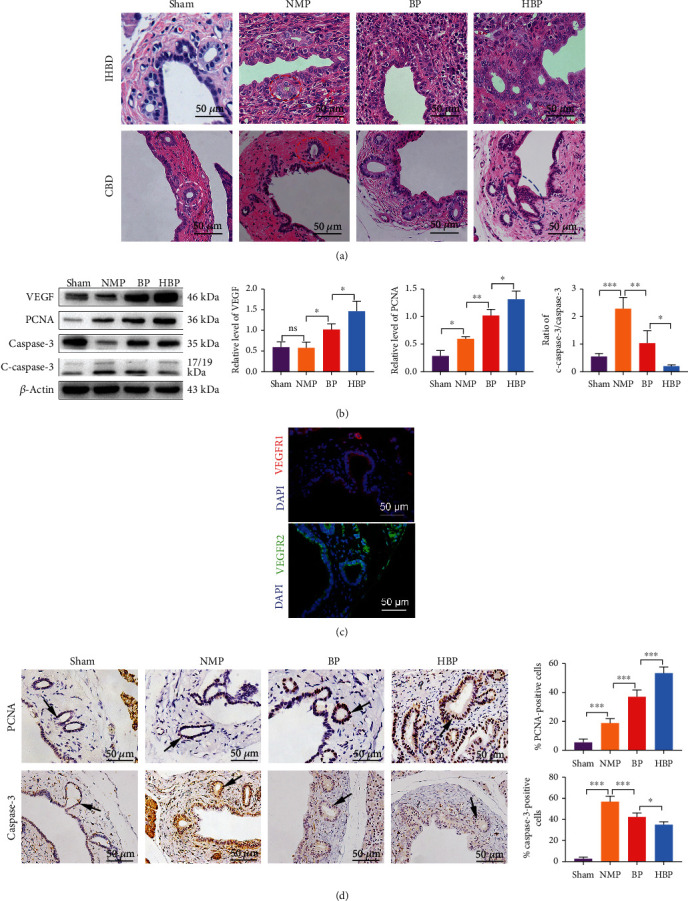
Transformation of PBGs from a static phenotype to an active phenotype. (a) The morphology of PBGs in the sham group was intact, and cells were round with large and round nuclei located in the center of the cells (white ellipse). The morphology of PBGs in the NMP group was destroyed (red ellipse), and continuity was broken, with necrotic cells. PBGs were relatively well preserved in the BP and HBP groups, which were expanded, and a large number of proliferating cells appeared at the connecting tubes between the PBGs and the lumen of the bile ducts (blue ellipse). (b) Relative protein levels of VEGF, PCNA, caspase-3, and cleaved caspase-3 in each group were detected using western blotting (*n* = 3). (c) Immunofluorescence staining showed that PBGs expressed VEGFR1 and VEGFR2. (d) Expression of PCNA and caspase-3 in each group was marked by immunohistochemistry staining. Compared with the sham group (5.50 ± 2.29%), the number of PCNA-positive cells was higher in the NMP group (18.90 ± 3.00%, *P* = 0.0002) and even higher in the BP group (37.10 ± 4.64%, *P* < 0.0001 vs. the NMP group). The increase in the HBP group was the most significant (53.50 ± 4.24%, *P* < 0.0001 vs. the BP group). Compared with the sham group (2.70 ± 1.48%), numbers of caspase-3-positive cells (56.90 ± 5.34%, *P* < 0.0001) were significantly increased in the NMP group and decreased in the BP group (42.30 ± 3.96%, *P* < 0.0001 vs. the NMP group) and further decreased in the HBP group (35.10 ± 2.66%, *P* = 0.0306 vs. the BP group). ^∗^*P* < 0.05, ^∗∗^*P* < 0.01, and ^∗∗∗^*P* < 0.001. PBG: peribiliary gland; NMP: normothermic machine perfusion; HBP: HO-1/BMMSCs plus NMP; BP: BMMSCs plus NMP; VEGF: vascular endothelial growth factor; PCNA: proliferating cell nuclear antigen; VEGFR1: vascular endothelial growth factor receptor 1; VEGFR2: vascular endothelial growth factor receptor 2.

Histology showed severe damage to PBGs, destruction of the intraluminal epithelium, punctate necrosis of biliary cells, and a small number of regenerative biliary cells in the NMP group (Figure 4(a)). In contrast, PBG cells were relatively well preserved, with a few signs of cell death, in the BP and HBP groups, with dilation of some PBGs, in which a large amount of regenerative biliary cells was observed, which migrated to the bile duct lumen through the connecting tube between the PBGs and the bile duct lumen (Figure 4(a)).

In addition, PBG cells in the HBP group expressed more VEGF (Figure 4(b)) after liver transplantation. PBGs also expressed vascular endothelial growth factor receptor 1 (VEGFR1) and receptor 2 (VEGFR2) (Figure 4(c)), indicating that VEGF secreted by PBGs promoted the growth of biliary cells by autocrine and paracrine pathways.

Caspase-3 expression was detected in PBGs and surrounding stromal cells after liver transplantation, indicating activation of the apoptosis pathway (Figure 4(b)). The number of caspase-3-positive PBG cells in the sham group (2.70 ± 1.48%) was significantly lower than that in the NMP group (56.90 ± 5.3%). In contrast, caspase-3-positive PBG cells decreased in the BP group (42.30 ± 3.96%, *P* < 0.05), while that in the HBP group decreased more significantly (35.10 ± 2.66%, *P* < 0.05) (Figure 4(d)). The ratio of activated caspase-3 (cleaved caspase-3 (c-caspase-3)) to caspase-3 was the lowest in the HBP group (Figure 4(b)).

Immunohistochemical staining showed that 5.50 ± 2.29% of PBG cells expressed PCNA in the sham group, which increased significantly in the NMP group (18.90 ± 3.00%, *P* < 0.05 vs. the sham group). The proportion increased further in the BP group (37.10 ± 4.64%, *P* < 0.05 vs. the NMP group) and increased more significantly in the HBP group (53.50 ± 4.24%, *P* < 0.05 vs. the BP group) (Figure 4(d)). These results indicated that the process of liver transplantation caused severe damage to bile duct epithelial cells as well as PBG cells, and HO-1/BMMSCs promoted the proliferation of residual PBG cells.

### 3.5. Transformation of PBG Cells to a Mature Phenotype during Proliferation and Regeneration

**Figure 5:**
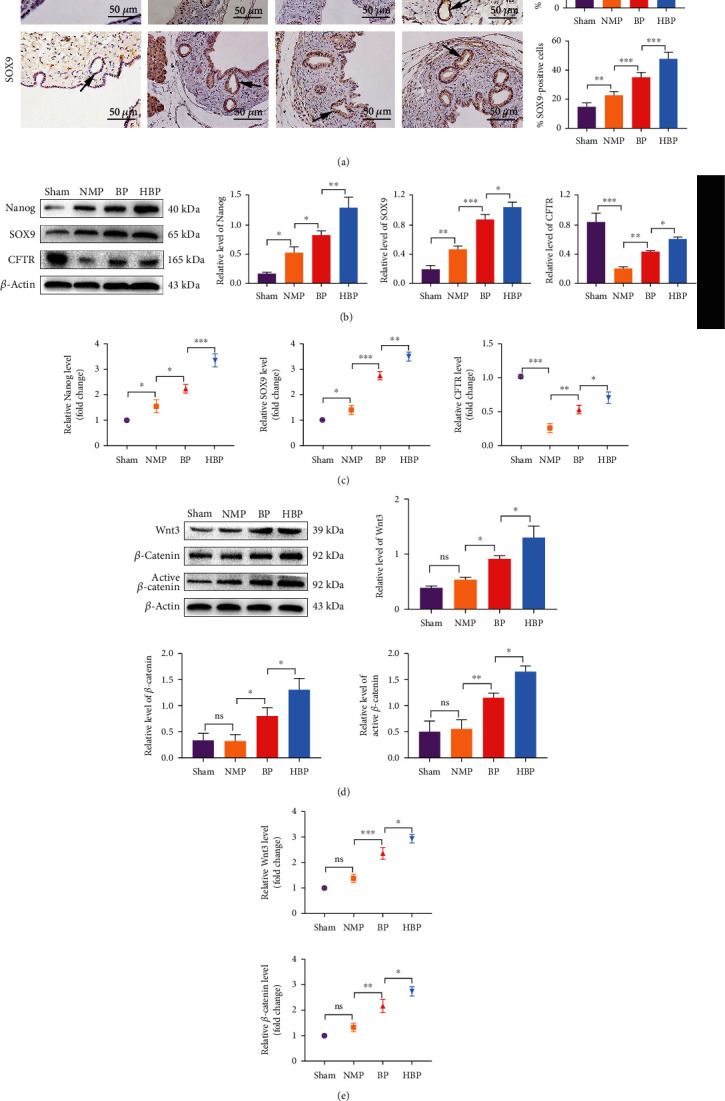
Increased stemness and activation of the Wnt signal pathway in PBGs. (a) Pluripotent cell marker- (Nanog- and SOX9-) positive cells in PBGs were marked by immunohistochemistry (arrow). Nanog- and SOX9-positive cells in the sham group were 15.10% ± 2.19 and 14.90% ± 2.56, respectively, slightly increased in the NMP group (35.00 ± 5.78%, *P* < 0.0001 vs. the sham group, 22.70 ± 2.44%, *P* = 0.0097 vs. the sham group) and significantly increased in the BP group (45.20 ± 4.44%, *P* = 0.00123 vs. the NMP group and 35.10 ± 3.32%, *P* = 0.0001 vs. the NMP group, respectively) and the HBP group (58.40 ± 4.88%, *P* = 0.0015 vs. the BP group and 47.70 ± 4.60%, *P* = 0.0001 vs. the BP group). Relative levels of Nanog, SOX9, and CFTR in each group were detected by western blotting (b) and PCR (c) (*n* = 3). (d) Western blotting analysis of Wnt3, total *β*-cat, and active-*β*-cat showed no statistical differences between the NMP group and the sham group, while expression of Wnt3, total *β*-cat, and active-*β*-cat increased in the BP and HBP groups (*n* = 3). (e) Relative levels of *Wnt3* and *Ctnnb*1 (total *β*-cat) levels were detected using PCR (*n* = 3). ^∗^*P* < 0.05, ^∗∗^*P* < 0.01, and ^∗∗∗^*P* < 0.001. PBG: peribiliary gland; NMP: normothermic machine perfusion; HBP: HO-1/BMMSCs plus NMP; BP: BMMSCs plus NMP; SOX9: SRY-box transcription factor 9; CFTR: cystic fibrosis transmembrane conductance regulator; *β*-cat: *β*-catenin.

To explore the maturation of proliferating and migrating PBG cells, we evaluated the expression of SOX9, homeobox protein Nanog, and CFTR. SOX9 is a marker of stem cells, Nanog is a transcription factor closely related to self-renewal of undifferentiated stem cells, and CFTR is a transporter on the surface of mature biliary cells. In the sham group, the proportions of PBG cells expressing SOX9 and Nanog were 15.10 ± 2.19% and 14.90 ± 2.56%, respectively (Figure 5(a)), suggesting the phenotype of endoderm progenitor cells. The proportion of SOX9- and Nanog-positive cells increased in the NMP group (35.00 ± 5.78% and 22.70 ± 2.44%, respectively) (Figures 5(a)–5(c)), suggesting proliferation of biliary progenitor cells. Compared with that in the NMP group, the proportion of cells expressing Nanog and SOX9 in the BP group (45.20 ± 4.44% and 35.10 ± 3.32%, respectively) and the HBP group (58.40 ± 4.88% and 47.70 ± 4.60%, respectively) further increased, and the increase in the HBP group was the most significant (Figures 5(a)–5(c)). The expression of Nanog and SOX9 in each group was consistent with that of PCNA, suggesting that BMMSCs and HO-1/BMMSCs promoted the proliferation of biliary progenitor cells in PBGs. Accordingly, expression of CFTR decreased significantly in the NMP group, indicating that a large number of mature bile duct cells were destroyed, while expression of CFTR increased in the BP and HBP groups (Figures 5(b) and 5(c)), suggesting that proliferated biliary progenitor cells had differentiated from a primitive pluripotent phenotype to mature biliary cells, replenishing the lost biliary epithelium.

### 3.6. The Wnt Signaling Pathway Triggered the Proliferation of PBG Cells and the Repair of Bile Duct Injury

We detected the expression of Wnt signaling pathway members Wnt3, *β*-catenin, and unphosphorylated *β*-catenin (active *β*-catenin). Results showed that expression levels of Wnt3, *β*-catenin, and active *β*-catenin in the BP and HBP groups were significantly higher than those in the sham and NMP groups (Figures 5(d) and 5(e)).

**Figure 6:**
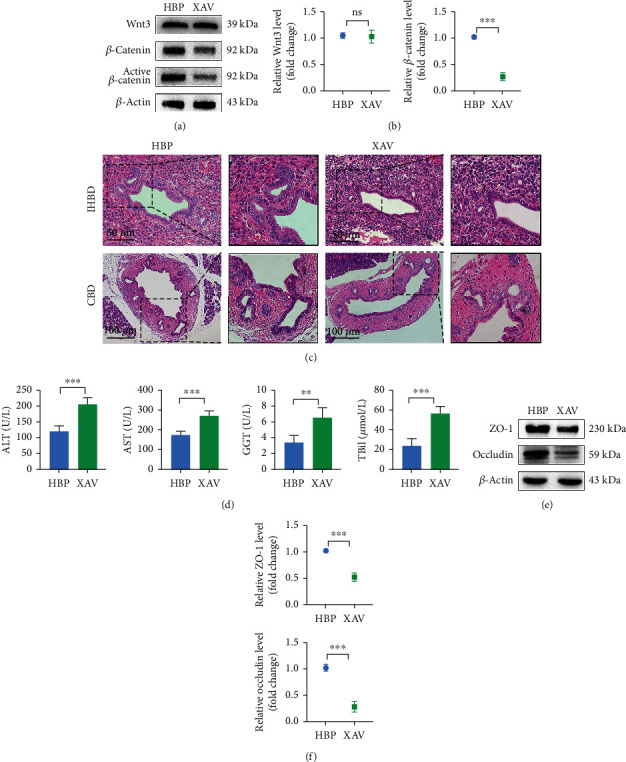
Wnt signal pathway triggered proliferation of biliary progenitor cells to repair bile duct injury. Activation levels of the Wnt signal pathway in each group were detected by western blotting (a) and PCR (b) (*n* = 3). (c) Histological changes of bile ducts and PBGs after administration of XAV-939. (d) Liver function changes after administration of XAV-939. ALT, AST, GGT, and TBil in the HBP group were 120.70 ± 17.96 U/L, 176.40 ± 18.61 U/L, 24.12 ± 7.26 U/L, and 3.42 ± 0.93 *μ*mol/L, respectively, while they were worse in the XAV-939 group (207.40 ± 20.41 U/L, 273.8 ± 24.11 U/L, 56.92 ± 7.32 U/L, and 6.58 ± 1.26 *μ*mol/L, respectively). Relative levels of tight junctions (ZO-1 and occludin) in each group were detected using western blotting (e) and PCR (f) (*n* = 3). ^∗^*P* < 0.05, ^∗∗^*P* < 0.01, and ^∗∗∗^*P* < 0.001. PBG: peribiliary gland; ALT: alanine aminotransferase; AST: aspartate aminotransferase; GGT: gamma-glutamyl transpeptidase; TBil: total bilirubin; HBP: HO-1/BMMSCs plus NMP.

**Figure 7:**
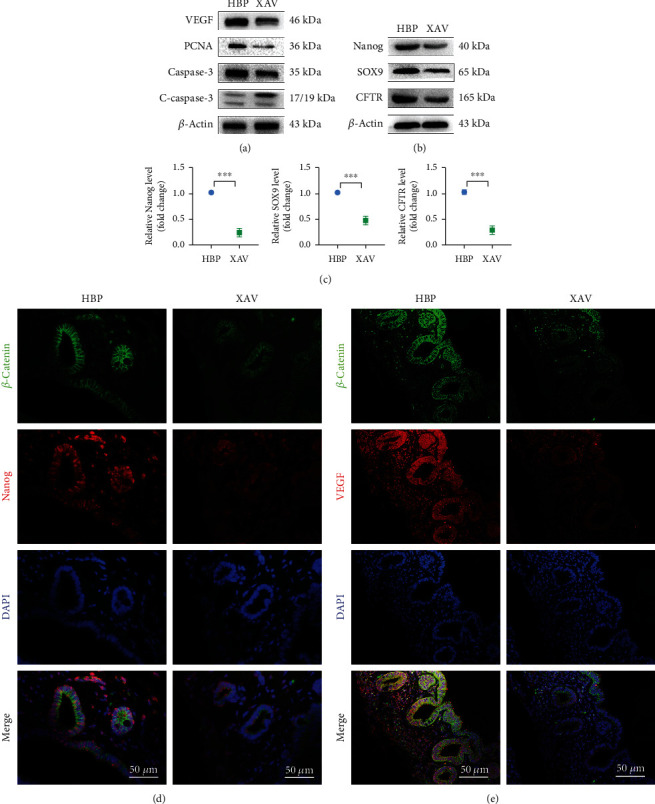
Changes in the proliferative activity and stemness of PBG cells after inhibition of the Wnt signal pathway. (a) Relative levels of VEGF, PCNA, caspase-3, and cleaved caspase-3 in each group were detected using western blotting (*n* = 3). Relative levels of Nanog, SOX9, and CFTR in each group were detected by western blotting (b) and PCR (c) (*n* = 3). Immunofluorescence staining showed that XAV-939 effectively degraded *β*-catenin and downregulated expression of Nanog (d) and VEGF (e) at the same time. ^∗^*P* < 0.05, ^∗∗^*P* < 0.01, and ^∗∗∗^*P* < 0.001. PBG: peribiliary gland; VEGF: vascular endothelial growth factor; PCNA: proliferating cell nuclear antigen; SOX9: SRY-box transcription factor 9; CFTR: cystic fibrosis transmembrane conductance regulator.

Administration of XAV-939 (an inhibitor of Wnt signal pathway) *in vivo* significantly reduced expression levels of *β*-catenin and active *β*-catenin (Figures 6(a) and 6(b)) and inhibited reparative effect of HO-1/BMMSCs. Compared with that in the HBP group, histology showed that biliary epithelial injury was more severe and PBG cell proliferation was decreased in the XAV group (Figure 6(c)); accordingly, liver function (Figure 6(d)) and tight junctions (Figures 6(e) and 6(f)) were worse. In the XAV group, the expression levels of VEGF and PCNA decreased, while those of caspase-3 and c-caspase-3 increased (Figure 7(a)). The expression levels of Nanog, SOX9, and CFTR also decreased (Figures 7(b) and 7(c)). Immunofluorescence staining showed that XAV-939 effectively degraded *β*-catenin, thereby inhibiting the classical Wnt signaling pathway and reducing expression of Nanog and VEGF (Figures 7(d) and 7(e)), i.e., both the growth and stemness of PBG cells were inhibited. When the Wnt signaling pathway was inhibited, HO-1/BMMSCs could not fully play their role in damage repair, which suggested that HO-1/BMMSCs might promote the proliferation and differentiation of biliary progenitor cells by activating the Wnt signaling pathway (Fig. S3).

## 4. Discussion

DCD livers suffer from severe IRI. Livers lose oxygen and nutrient supply after circulatory arrest, resulting in ATP depletion and massive accumulation of metabolic waste. In addition, oxygen influx during reperfusion leads to the production of reactive oxygen species, extensive activation of inflammatory pathways, and cell death. IRI is an important factor causing bile duct complications after transplantation (14), which can lead to graft dysfunction and the need for retransplantation in severe cases. Therefore, under this premise, we have developed a new method showing the potential to reduce biliary injury caused by ischemia-reperfusion in rodents.

BMMSCs have functions of immunomodulation and tissue repair, prompting widespread interest in their use in the field of organ transplantation [17, 18]. However, BMMSC application still faces two unresolved and unavoidable problems. First, although BMMSCs can migrate to the injured site guided by chemokines, a large number of BMMSCs are trapped in lung capillaries after intravenous administration [19, 20], resulting in relatively few cells reaching the injured site. Second, BMMSCs engrafted in recipients will be cleared in a short time, usually no more than 24 hours [21].

To solve these two problems, we optimized BMMSC therapy in two ways. First, we combined cell therapy with NMP to increase the engraftment rate of BMMSCs in livers. In our previous study, we established an NMP system and verified that it protected liver grafts from IRI compared with static cold storage and observed that no extra injury was added by the NMP system [13]. NMP simulates the physiological environment of the liver, washing off metabolic waste; replenishing oxygen, nutrients, and metabolic substrates; and maintaining normal metabolism of livers to the maximum extent [22]. In addition to this protective effect on liver grafts, we used NMP as a method of infusion of BMMSCs through the portal vein. Compared with conventional intravenous administration, this new strategy increased the engraftment rate of BMMSCs in livers and improved the quality of DCD livers during *ex situ* preservation. Second, to solve the problem of the short survival time of BMMSCs in the host, we overexpressed heme oxygenase-1 (HO-1) in BMMSCs. HO-1 is a rate-limiting enzyme catalyzing the conversion of heme to biliverdin, which has antioxidant and anti-inflammatory functions [23], and could be used as a cytoprotective molecule [24]. We used this strategy to effectively prolong the survival time of BMMSCs in livers. Our study demonstrated that HO-1/BMMSCs were superior to BMMSCs in terms of liver function, histology, and survival time of the recipients.

We established a rat model of DCD liver transplantation in which a massive biliary epithelium loss of extrahepatic and large intrahepatic bile ducts occurred because of severe ischemia-reperfusion injury. After administration of HO-1/BMMSCs, proliferation of biliary progenitor cells in PBGs and restoration of the bile duct IRI after DCD liver transplantation were observed. PBG cells were activated after bile duct injury to repair the injured biliary epithelium, which is consistent with results in other models of bile duct disease [11, 25]. These studies and the present study suggested that PBGs function as a depository of biliary progenitor cells for epithelial regeneration after injury. PBGs are tiny recesses distributed along the large intrahepatic and extrahepatic bile ducts, which secrete mucus and enzymes to maintain the physiological functions of the biliary epithelium [26]. They also function as a niche of biliary progenitor cells, maintaining the renewal and regeneration of the biliary epithelium [27]. The degree of PBG injury is proportional to the incidence of bile duct stricture after liver transplantation [28], suggesting that the structural and functional integrity of PBGs is an important guarantee for the repair of bile duct injury. Our study validates the core role of PBGs in the repair of severe bile duct injury and provides new insights into the repair pathway and mechanism of bile duct injury after liver transplantation.

We found that HO-1/BMMSCs promoted the proliferation of PBGs and inhibited cell apoptosis. Normally, PBGs are small acini arranged in clusters. After IRI in the liver transplantation model, most of the PBGs were destroyed, and few of them were dilated. PBGs presented more complete shapes and showed more obvious dilation after administration of HO-1/BMMSCs. The phenomenon of PBG dilatation might be caused by high levels of cell proliferation. After the administration of HO-1/BMMSCs, PBG cells expressed high levels of VEGF, and PBGs also expressed VEGF receptors. Therefore, VEGF secreted by PBGs not only promotes the proliferation of neighboring vascular endothelial cells but also has autocrine and paracrine effects on PBG cells, increasing their proliferation and inducing the transformation from a static phenotype to an active phenotype. This phenomenon indicates that the growth factor VEGF is also related to proliferation of biliary cells [29]. We found that HO-1/BMMSCs were associated with not only the proliferation of PBG cells but also the increased proportion of pluripotent cells in PBGs. Currently, there is no clear evidence as to how these changes occur; therefore, further in-depth studies are required.

Wnt signaling plays a vital role in animal embryonic development, tissue regeneration, and other physiological processes. Wnt is a secretory protein that transmits signals between cells. When the Wnt signaling pathway is activated, *β*-catenin accumulates in the cytoplasm and then enters the nucleus to act as a transcription factor [30]. The Wnt signaling pathway controls the growth and destiny of stem cells. Activation of the Wnt pathway increases the number of adult stem cells, while retaining their differentiation potential and maintaining self-renewal [31]. Nanog is one of the main proteins responsible for maintaining the pluripotency of stem cells and is only expressed in pluripotent cells [32]. During activation of the Wnt pathway, *β*-catenin acts as a transcription factor to upregulate *Nanog* expression [33, 34]. *VEGF* is also one of target genes of the Wnt pathway, and *β*-catenin regulates its expression by binding to its promoter [35, 36]. We found that HO-1/BMMSCs were associated with activation of the Wnt pathway, corresponding to the proliferation and pluripotency of biliary progenitor cells. Proliferative biliary progenitor cells migrate to the injured site and differentiate from a primitive pluripotent phenotype to a mature biliary epithelial cell phenotype after leaving the stem cell niche maintained by Wnt, which was verified by the increased expression of CFTR after the administration of HO-1/BMMSCs.

Previously, it was thought that mesenchymal stem cells (MSCs) migrated to the injured site and differentiated into functional cells to repair damaged tissue [37]. However, few MSCs engrafted into target organs after intravenous administration, which is insufficient to repair the damage [20], which suggested that MSCs do not perform a tissue repair function through differentiation. In the 1970s, Dexter et al. [38] used bone marrow-derived adherent cells to successfully maintain the self-renewal, proliferation, and differentiation potential of hematopoietic stem cells. These bone marrow-derived adherent cells, known today as BMMSCs, are also known as bone marrow stromal cells. Studies by Dexter et al. suggested that BMMSCs provide the necessary microenvironment for self-renewal and differentiation of pluripotent cells. BMMSCs might alter the tissue microenvironment in a paracrine manner. BMMSCs secrete a large number of active factors that promote proliferation, stimulate angiogenesis, and inhibit apoptosis and inflammation [39]. Notably, these factors also maintain the pluripotency and stimulate the proliferation of endogenous stem cells and progenitor cells to repair injury [40]. We hypothesized that BMMSCs affected the proliferation and differentiation of biliary progenitor cells by activating the Wnt signaling pathway in the biliary microenvironment, which supports the above view. Therefore, the ability of BMMSCs to secrete active factors and cytokines to alter the tissue microenvironment might be an important mechanism of tissue repair.

BMMSCs might enhance two stem cell properties of PBG cells through Wnt signaling: pluripotency, characterized by a positive Nanog expression, and the ability to proliferate extensively, as observed in liver transplantation models where biliary progenitor cells rapidly proliferate and differentiate to restore the biliary epithelium. In this study, we provided evidence supporting the view that optimized BMMSCs promoted the proliferation, migration, and maturation of endogenous biliary progenitor cells in PBGs by activating the Wnt pathway to repair bile duct injury after liver transplantation. This finding provides evidence for the pathophysiology of bile duct injury repair in DCD liver transplantation and suggests a new way to expand the safe application of DCD liver transplantation. However, interpretation and translation of the results to the clinical setting should be carefully considered. Biliary complications occur in humans usually after several months following transplantation, but we observed the histology just 7 days after surgery. Although the survival analysis was sufficiently long, we did not clarify the cause of death of animals. Additional study is needed to prove whether the novel strategy effectively reduces the occurrence of biliary complications after transplantation, improves quality of DCD livers, and solves the problem of donor pool shortage. Additionally, results from this rodent model may not be directly translated to humans and would need to be verified. Overall, we believe that our results provide a new insight into the pathophysiology of bile duct injury in liver transplantation and present new opportunities to expand the safe utilization of DCD liver grafts.

## 5. Conclusion

We successfully established an improved method of stem cell therapy combined with mechanical perfusion to preserve organs. NMP increased the engraftment of BMMSCs in livers, and transfection with the gene encoding HO-1 prolonged the survival time of BMMSCs in the recipients. HO-1/BMMSCs combined with NMP showed the potential to repair bile duct IRI and improve prognosis in a rat DCD liver transplantation model. The mechanism might be that HO-1/BMMSCs activate the Wnt pathway to maintain the pluripotency of biliary progenitor cells in PBGs to drive their proliferation, migration, and maturation. This study provides a valuable experimental basis to improve the quality of DCD livers, reducing postoperative biliary complications, and improving the long-term survival rate.

## Data Availability

The data used to support the findings of this study are included within the article.

## Abbreviations

ALT: Alanine aminotransferase
AST: Aspartate aminotransferase
BMMSCs: Bone marrow mesenchymal stem cells
BP: BMMSCs plus NMP
*β*-cat: *β*-Catenin
CFTR: Cystic fibrosis transmembrane conductance regulator
DCD: Donors after circulatory death
GGT: Gamma-glutamyl transpeptidase
HO-1: Heme oxygenase-1
HBP: HO-1/BMMSCs plus NMP
NMP: Normothermic machine perfusion
PBG: Peribiliary gland
PCNA: Proliferating cell nuclear antigen
SOX9: SRY-box transcription factor 9
TBil: Total bilirubin
VEGF: Vascular endothelial growth factor
VEGFR1: Vascular endothelial growth factor receptor 1
VEGFR2: Vascular endothelial growth factor receptor 2.

## Supplementary Materials

Supplementary Materials Table S1: primers for qRT-PCR. Figure S1: diagram of NMP. Figure S2: morphology and characteristics of HO-1/BMMSCs. (A) HO-1/BMMSCs were adherent cells with long spindle shape. (B) HO-1/BMMSCs could differentiate into osteoblasts *in vitro*. (C) HO-1/BMMSCs could differentiate into adipocytes *in vitro*. (D–F) HO-1/BMSCs were positive for CD29, CD90, and RT1-A and negative for CD34, CD45 and RT1-B using flow cytometry. (G, H) Western blotting and PCR confirmed HO-1 overexpression in HO-1/BMMSCs. (I) HO-1/BMMSCs transfected with adenovirus vectors expressing GFP in the fluorescence field. Figure S3: schematic diagram of mechanism underlying HO-1/BMMSCs modulating PBG cells to repair injury.
