# Supplementary material for: Heme Oxygenase-1-Modified Bone Marrow Mesenchymal Stem Cells Combined with Normothermic Machine Perfusion Repairs Bile Duct Injury in a Rat Model of DCD Liver Transplantation via Activation of Peribiliary Glands through the Wnt Pathway
Source: Stem Cells Int. 2021 Jul 1;2021:9935370. doi: 10.1155/2021/9935370 (PMC8275434; doi:10.1155/2021/9935370)
Supplement: Supplementary Materials — Table S1: primers for qRT-PCR. Figure S1: diagram of NMP. Figure S2: morphology and characteristics of HO-1/BMMSCs. (A) HO-1/BMMSCs were adherent cells with long spindle shape. (B) HO-1/BMMSCs could differentiate into osteoblasts in vitro. (C) HO-1/BMMSCs could differentiate into adipocytes in vitro. (D–F) HO-1/BMSCs were positive for CD29, CD90, and RT1-A and negative for CD34, CD45 and RT1-B using flow cytometry. (G, H) Western blotting and PCR confirmed HO-1 overexpression in HO-1/BMMSCs. (I) HO-1/BMMSCs transfected with adenovirus vectors expressing GFP in the fluorescence field. Figure S3: schematic diagram of mechanism underlying HO-1/BMMSCs modulating PBG cells to repair injury. [file 9935370.f1.pdf]

Supplementary Table

Table. 1 Primers for qRT-PCR

Gene	Primer sequence
β-catenin	F 5’-TACCGCTGGGACCCTACACAAC-3’
β-catenin	R 5’-GCGTGGTGATGGCGTAGAACAG-3’
Wnt3	F 5’-CAGCCTGACTTCCGAGCCATTG-3’
Wnt3	R 5’-ACTCCCGATGCTTCTCCACCAC-3’
CFTR	F 5’-AGTGTCTTCCAGCGAGCCCTTAG-3’
CFTR	R 5’-TCCTCCGTCTCCTCTTTCACAGC-3’
SOX9	F 5’-TGGCAGAGGGTGGCAGACAG-3’
SOX9	R 5’-CGTTGGGCGGCAGGTATTGG-3’
Nanog	F 5’-CTGCCTCTCCTCCGCCTTCC-3’
Nanog	R 5’-CTCGTCAGCCTCGGGACCAG-3’
VEGF	F 5’-CACGACAGAAGGGGAGCAGAAAG-3’
VEGF	R 5’-GGCACACAGGACGGCTTGAAG-3’
ZO-1	F 5’-CGCAGCCAGTTCAAACAAAGTTCC-3’
ZO-1	R 5’-GCAACATCAGCAATCGGTCCAAAG-3’
Occludin	F 5’-CAACGGCAAAGTGAATGGCAAGAG-3’
Occludin	R 5’-TCATCCACGGACAAGGTCAGAGG-3’
HO-1	F 5’-CAGACAGAGTTTCTTCGCCAGAGG-3’
HO-1	R 5’-TGTGAGGACCCATCGCAGGAG-3’
β-actin	F 5’-CGCGAGTACAACCTTCTTGC-3’
β-actin	R 5’-ATACCCACCATCACACCCTG-3’

Supplementary Figures

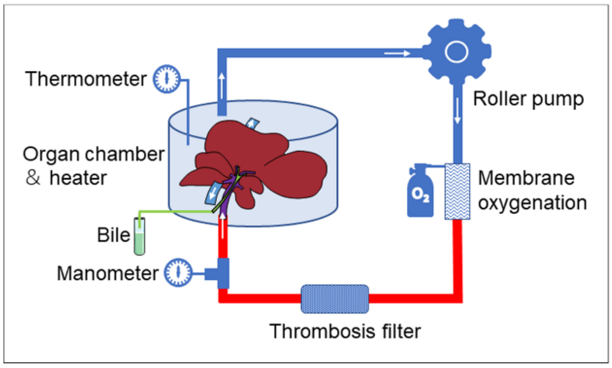

Supplementary Fig. 1 Diagram of NMP. The perfusion machine consists of an organ chamber, a rolling pump, a membrane oxygenator, a thrombus filter, a temperature sensor, and a pressure sensor. NMP; normothermic machine perfusion**.**

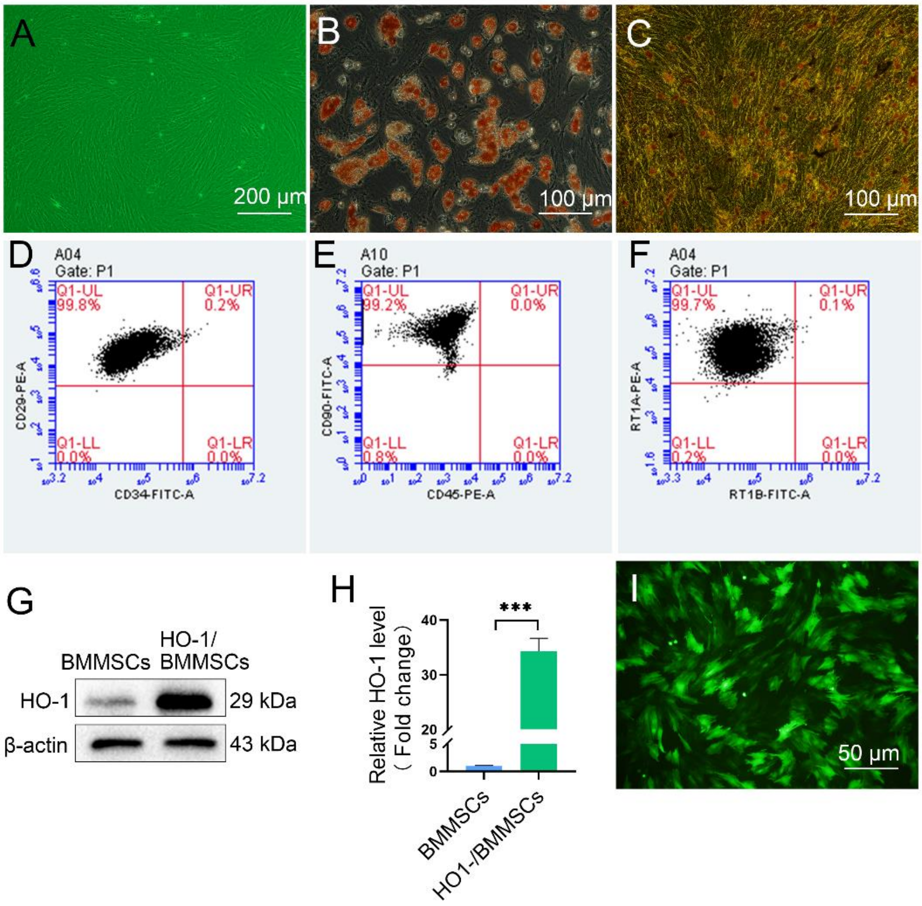

Supplementary Fig. 2 Morphology and characteristics of HO-1/BMMSCs. **A** HO-1/BMMSCs were adherent cells with long spindle shape. **B** HO-1/BMMSCs could differentiate into osteoblasts *in vitro*, and black calcium deposition was detected by Von Kossa staining. **C** HO-1/BMMSCs could differentiate into adipocytes; red lipid droplets were detected using oil red O staining. **D-F** HO-1/BMSCs were positive for CD29, CD90 and RT1-A and negative for CD34, CD45 and RT1-B using flow cytometry. Biological characteristics of HO-1/ BMSCs were not altered. **G, H** Western blotting and PCR confirmed HO-1 overexpression in HO-1/BMMSCs. **I** Overexpression of GFP in HO-1/BMMSCs. *P < 0.05, **P < 0.01, ***P < 0.001. BMMSCs, bone marrow mesenchymal stem cells; HO-1, heme oxygenase 1; CD29, integrin subunit beta 1; CD90, Thy-1 cell surface antigen; RT1-A, Rat MHC class I antibody; CD34, cluster of differentiation 34; CD45, protein tyrosine phosphatase receptor type C**;** RT-1B, Rat MHC class II antibody**;** GFP, green fluorescent protein.

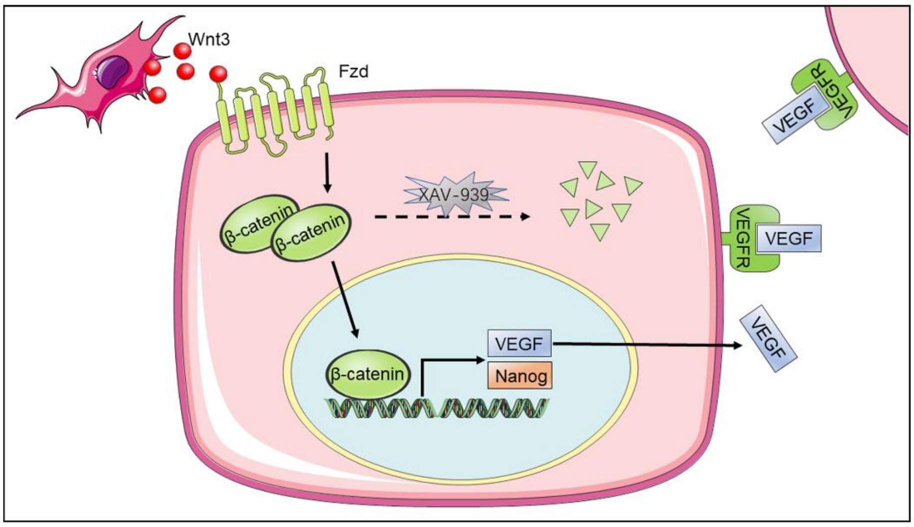

Supplementary Fig. 3 Schematic diagram of mechanism underlying HO-1/BMMSCs modulating PBG cells to repair injury. HO-1/BMMSCs activated the Wnt signal pathway, and β-catenin accumulated in the cytoplasm and then entered nucleus as a transcription factor to bind to promoter regions of *Vegf* and *Nanog* and upregulated their expression. Nanog maintained pluripotency of biliary progenitor cells, and VEGF has autocrine and paracrine effects on PBGs cells. XAV-939 degraded β-catenin and inhibited the effect of HO-1/BMMSCs. BMMSCs, bone marrow mesenchymal stem cells; HO-1, heme oxygenase 1; PBG, peribiliary gland; VEGF, vascular endothelial growth factor.
